# Development of Traditional Chinese Medicine in combination with EGFR Inhibitors against Cancer

**DOI:** 10.7150/jca.109420

**Published:** 2025-06-05

**Authors:** Xiao Chen, Tiansheng Zheng, Bingjie Hao, Shumeng Lin, Liduo Yue, Lihong Fan

**Affiliations:** 1 Department of Oncology, School of Medicine, Nantong University, Nantong, China.; 2 Institute of Energy Metabolism and Health, Shanghai Tenth People's Hospital, Tongji University School of Medicine, Shanghai, China.; 3 Department of Respiratory Medicine, Shanghai Tenth People's Hospital, Tongji University School of Medicine, Shanghai, China.; 4 Department of Pulmonology, Sixth People's Hospital Affiliated to Shanghai Jiao Tong University, China.

**Keywords:** EGFR, EGFR-TKI, EGFR monoclonal antibody, traditional Chinese medicine monomers, traditional Chinese medicine, cancer

## Abstract

Epidermal growth factor receptor (EGFR) is one of the most important and therapeutically significant targets in most cancer treatments, and EGFR-targeted therapy is widely performed to treat various tumors, such as non-small cell lung cancer. Although EGFR-targeted therapy has fewer side effects than conventional chemotherapy, they have limited applications and are prone to drug resistance. Traditional Chinese medicine (TCM), are promising solutions to address these challenges because of their biological activities, such as inhibition of EGFR-related signaling pathways, reversal of drug resistance, and mitigation of side effects of targeted therapy. Moreover, TCM is characterized by multiple targets, few side effects and good therapeutic effect Here, we summarized several typical traditional Chinese medicine monomers derived from traditional Chinese medicine that can be used along with EGFR inhibitors, as well as herbal plants with potential for natural product development and TCM. We focused on their mechanisms of action underlying the reversal of drug resistance, enhancement of drug efficacy, and mitigation of side effects; we also assessed relevant clinical studies that are available. However, the potential adverse effects (e.g., drug-drug interactions, hepatotoxicity, and immune-related side effects) of TCMs along with EGFR inhibitors need to be further investigated. This article provided new perspectives on the use of TCMs in EGFR-targeted therapy and emphasized the importance of safety assessment in future studies.

## Introduction

Cancer is a major social, public health, and economic issue of the 21^st^ century, with nearly one-sixth (16.8%) of global deaths and a quarter (22.8%) of deaths due to noncommunicable diseases (NCDs). Studies suggest that about one-fifth of men or women will suffer from cancer in their lifetime. By 2022, about one-ninth of men and one-twelfth of women were predicted to have died due to cancer, and nearly 20 million new cases of cancer developed worldwide. The estimated number of deaths due to cancer worldwide is 9.7 million 1. These data suggest that cancer is a major global problem affecting humans. In China and most developed countries, cancer is becoming the leading cause of death, and deaths due to cancer have surpassed those due to cardiovascular disease 2. By 2022, about 4,824,700 new cancer cases and 2,574,200 new cancer-related deaths were expected in China 3. Cancer is also a major medical and health problem in China, as it hinders the development of the economy and the health of people.

Many patients with cancer display strong EGFR expression levels, abnormal posttranslational modifications, and even sequence mutations 4, 5. Therefore, EGFR is one of the most promising and challenging targets for addressing cancer 6. Although there are fewer side effects of EGFR-targeted therapy than traditional chemotherapy, it is suitable for fewer people and is prone to developing drug resistance 7. Hence, EGFR-targeted therapy faces many challenges.

Traditional Chinese medicine monomers have a high diversity, and the majority of them originate from herbal plants that have been used in Traditional Chinese Medicine for a long time. TCM application involves using natural products with different pharmacological effects to prevent and treat diseases. The application of TCM is ubiquitous 8. Since the development of medicine, many natural products including traditional Chinese medicine monomers or their analogs, such as paclitaxel, have been used in clinical practice because of their anticancer properties 8, 9. Many types of TCMs, such as the Chinese herbal Fuzheng Yiai Decoction, are used as supplements, conditioning methods, or adjuvant treatments in clinical antitumor treatment plans. 10, 11. Therefore, they can be used as drugs or supplemental adjuncts to address the challenges associated with EGFR treatment techniques. Based on the current research on EGFR-targeted therapy, we summarized several traditional Chinese medicine monomers and compound prescriptions that produce positive effects in combination with EGFR inhibitors (EGFRIs).

## Epidermal growth factor receptor signal transduction pathway

**Figure 1:**
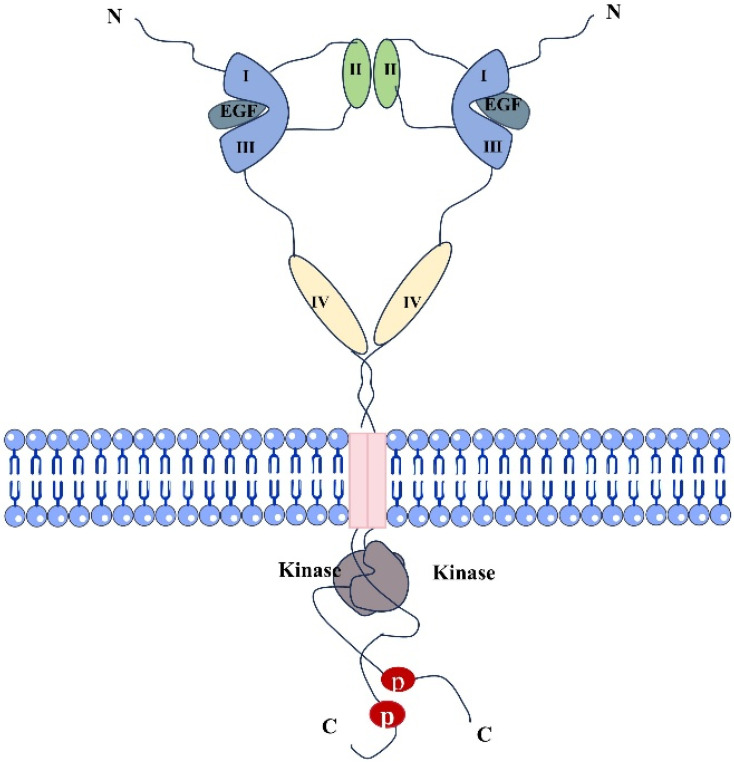
Structural diagram of dimers formed by EGFR and EGF ligand binding.

**Figure 2:**
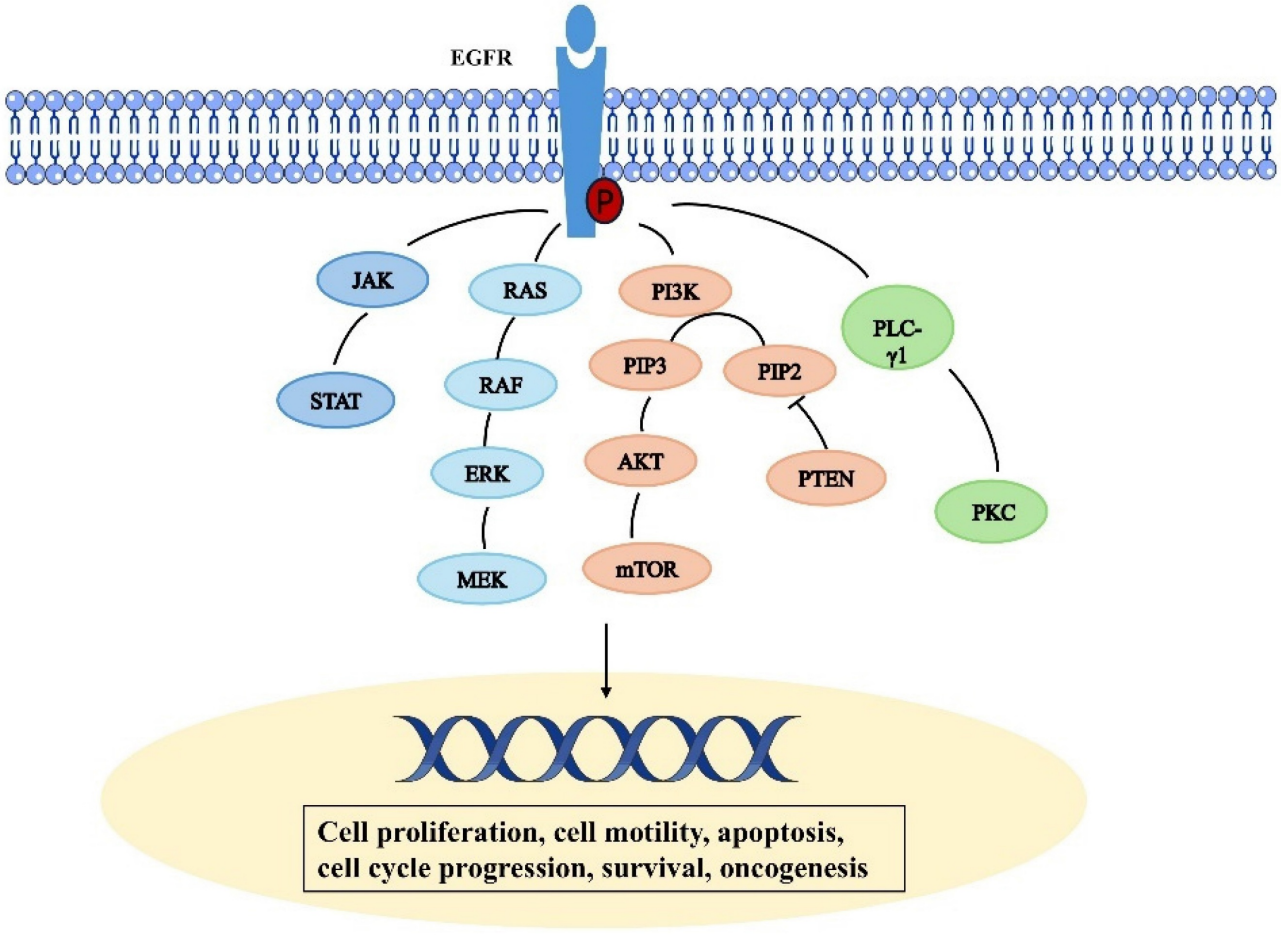
Schematic representation depicting the epidermal growth factor receptor signal transduction pathway.

Epidermal growth factor receptor (EGFR) is a tyrosine kinase receptor in the ErbB family 12. It is a transmembrane glycosylated protein, that includes an extracellular domain, a transmembrane domain, and an intracellular domain (Fig. 1). Its extracellular domain consists of subregions, I, II, III, and IV; the II domain is involved in the formation of homodimers and heterodimers. The transmembrane domain is a hydrophobic region anchored to the cell membrane, and consists of 19-25 amino acid residues. The intracellular region is the carboxy-terminal region of the cytoplasm with a protein kinase domain, which consists of about 550 amino acid residues and contains three subregions, including the tyrosine kinase region (TK), the near membrane region (JM), and the C-terminal region (CTD). When EGFR and its ligands dimerize and are activated, ATP binds to the tyrosine kinase region and activates downstream signaling pathways 13, 14. The near-membrane region can regulate kinase dimerization and has a moderation effect on downstream signaling pathways. The C-terminal domain facilitates self-phosphorylation when EGFR is activated, and phosphorylated residues activate intracellular signaling pathways 15. It participates in the occurrence and metastasis of related cancers, demonstrating a moderation role in these pathways 6. The relevant pathways are summarized in Figure 2. Human EGFR is encoded by two transcripts of 10.5 kb and 5.8 kb (isoform A) 16. Except for full-length EGFR, the other isoforms are generated by alternative RNA splicing. Along with these two transcripts that generate the full-length EGFR isoform, the EGFR gene also produces three alternative transcripts that are 1.8, 2.4, and 3.0 kb in size and encode isoforms C, B, and D, respectively 17. The 3.0 kb isoform D is associated with cell membranes and can be detected in human serum 18. Studies and methodological tools for the application of these subtypes to the treatment and diagnosis of diseases have progressed modestly but need to be further investigated 19.

### RAS/RAF/mitogen-activated protein kinase pathway

EGF stimulates the activation of the downstream RAS and increases its GTP-binding state (Ras-GTP), thereby linking the RAS to upstream receptor tyrosine kinase (RTK) signaling 20. Adaptor proteins such as Grb2 recognize the sequence homology 2 (SH2) domain and thus recruit guanine nucleotide exchange factors (GEF) such as SOS-1 or CDC25 to the cell membrane. GEF facilitates conformational changes and the exchange of GDP and GTP via interacting with Ras proteins at the cell membrane. Raf is drawn to the cell membrane by binding to the Ras switch I domain and lipid binding when Ras is activated 21. When RAF protein kinase activity is activated by activated Ras, RAF kinase is phosphorylated, which in turn activates MEK (serine/tyrosine/threonine kinase). The mitogen activated protein kinase ERK is activated by MEK phosphorylation, and when ERK is phosphorylated, its kinase activity is triggered as well as several downstream targets involved in the control of cell proliferation are phosphorylated. Resistance to EGFR treatment is linked to abnormal RAS protein activity 22.

### Phosphatidylinositol triphosphate (PI3K) and serine-threonine protein kinase (AKT) pathways

PI3K/Akt/mTOR signaling is one of the important signaling pathways regulating cellular function, and PI3K/Akt/mTOR hyperactivation is seen in most solid tumors and is one of the most common dysregulated signals found in cancer patients 23. PI3K/Akt/mTOR signaling pathway is regulated by phosphatase and tensin homologs (PTEN) that are missing on chromosome 10 and human epidermal growth factor receptor (EGFR) 24. When cytosolic EGFR binds to ligands, cytoplasmic tyrosine kinases are activated, which leads to autophosphorylation and downstream conduction patency. This results in the PI3K/Akt chain reaction, which is the most important known EGFR-regulated transduction pathway to date 25. There are three types of PI3K, but class I PI3K is the most extensively researched. When PI3K is activated, a second messenger, PIP3, is produced on the plasma membrane. This messenger binds to the signal proteins AKT and PDK1 (phosphoinositide dependent kinase-1), which have PH domains in cells. This encourages PDK1 to phosphorylate Ser308 of the AKT protein, which in turn activates AKT. By phosphorylating downstream components such as a variety of enzymes, kinases, and transcription factors, activated AKT modulates cellular activity. mTOR is a mammalian target of rapamycin (mTOR), which is an important serine-threonine protein kinase downstream of PI3K/Akt 26. It regulates the growth, survival, and invasion and metastasis of tumor cells by activating ribosomal kinases.

### Signal transducer and activator of transcription (STAT) pathway

STAT is a nuclear transcription factor. However, STAT is found in the cytoplasm of cells that are at rest. Following activation, STAT molecules dimerize and go into the cell's nucleus. STAT proteins exist as inactive monomers. Through their SH2 domain, signal transducers and transcriptional activators (STAT-1, 3, and 5) interact with EGFR; nevertheless, their transcriptional activity remains non-functional until EGFR phosphorylation occurs 27. EGFR can regulate the STAT signaling pathway through two different mechanisms: JAK dependent and JAK independent. EGFR stimulation induces Tyr 701 phosphorylation of STAT 1, which further initiates the formation of complexes between STAT 1 and STAT 3 and JAK 1 and JAK 2 28. STAT3 plays a critical role in cancer development and abnormal activation is associated with several types of cancer, including brain, lung, pancreas, endometrium, colorectal, kidney, breast. It also plays an important role in resistance to tyrosine kinase inhibitors 29.

### PLC-γ1 pathway

The binding of ligands to EGFR leads to phosphorylation of PLC - γ 1 tyrosine residues and activation of lipase. Phosphatidylinositol-specific phospholipase C (PLC) plays an important role in transmembrane signaling 30. In response to extracellular stimuli such as hormones, growth factors, and neurotransmitters, PLC hydrolyzes phosphatidylinositol-4,5 bisphosphate (PIP2) and produces the second messenger's inositol 1,4,5 triphosphate (IP3) and diglycerides (DAG) 31. In addition, DAG activates protein kinase C (PKC) and IP 3, which induce the release of calcium from intracellular storage. These secondary messengers trigger a series of molecular interactions and alter the physiological state of cells 32.

## Epidermal growth factor receptor inhibitors approved for clinical practice

The main epidermal growth factor receptor inhibitors (EGFRIs) used in clinical practice are EGFR-TKIs and EGFR monoclonal antibodies. Specific monoclonal antibodies can bind to the extracellular domains II and III of EGFR. This type of antibody has several advantages. First, owing to its strong binding ability, it blocks the binding of ligands to EGFR, thereby inhibiting the activation of EGFR 33; Second, owing to its binding site in the extracellular region, EGFR can effectively target mutations in both wild-type and intracellular tyrosine kinase activity regions 34.

### Epidermal growth factor receptor tyrosine kinase inhibitor (EGFR-TKI)

The most commonly used drugs in clinical practice are tyrosine kinase inhibitors (TKIs) that target the EGFR activation domain. Moreover, many EGFR-TKIs are undergoing clinical trials 35. Its main mechanism of action involves binding to the tyrosine kinase domain of EGFR and competitively inhibiting ATP binding, thereby blocking the phosphorylation of tyrosine residues and inhibiting signal transduction 36. There are four generations of EGFR-TKIs approved for clinical use. Erlotinib and gefitinib are the first-generation small molecule EGFR-TKIs used to treat non-small cell lung cancer 37, 38. EGFR inhibition is reversible, and when EGFR T790M mutates, resistance to first generation drugs occurs 39. The second-generation EGFR-TKI is an irreversible small molecule drug developed to address the issue of acquired resistance in the first generation of drugs, its clinical efficacy is not satisfactory 40. The emergence of third-generation drugs can effectively solve the above problems and have acceptable clinical efficacy, but drug resistance quickly emerges 5. Fourth-generation EGFR-TKIs have good therapeutic effects on various types of tumors and cancers with multiple mutations 41. Researchers from many countries are intensively developing methods to address the unpredictable and changing situation of cancer. However, the efficacy of EGFR-TKIs is limited by drug resistance and the adverse effects of treatment.

### Monoclonal antibodies targeting EGFR receptors

Monoclonal antibodies targeting EGFR receptors have been approved for clinical use. They have become an important part of drug therapy for early cancer patients 42. Multiple monoclonal antibody drugs have been approved for clinical practice, making EGFR targeted therapy more selective 43. Cetuximab was the first approved EGFR mAb by the FDA in the US. After preliminary clinical trials, cetuximab was approved for treating advanced cancer in 2004, and two years later it was approved for treating head and neck squamous cell carcinoma that did not meet surgical indications 44, 45. However, cetuximab has almost no therapeutic effect on advanced colorectal cancer patients with KRAS and other RAS family gene activation mutations 46, 47. After the emergence of cetuximab, panitumumab was approved for use by the FDA via fast track. It is the first fully humanized antibody that binds to the extracellular domain of EGFR with high affinity 48. Additionally, panitumumab is ineffective in patients with other RAS mutations, along with those with KRAS mutations in exon 2 49. Other monoclonal antibodies approved for clinical use include necitumumab, nimotuzumab, and amivantamab 50-52. Many researchers in the field of medicine are trying to develop new monoclonal antibodies to address the complex cancer situation. However, monoclonal antibodies can be ineffective due to KRAS mutations and other RAS factor mutations or because of excessive accumulation of hyaluronic acid (HA) in the tumor microenvironment 53.

## Combination with approved EGFRIs

Two types of drugs used clinically to target EGFR for cancer treatment include monoclonal antibodies and EGFR-TKIs. The former binds to the extracellular domains II and III of EGFR, whereas EGFR-TKIs bind to the tyrosine kinase domain of EGFR 13. The latter is widely used. Owing to their low toxicity and minimal side effects, traditional herbs and their natural compounds have been studied and extracted by several researchers and have been applied in clinical practice to treat various diseases 54, 55. EGFR-TKIs or mAbs have been combines with other medications, such as various natural ingredients and formulations from TCM, in clinical practice and in several studies to produce a significantly greater effect than that achieved by its components when acting alone 56, 57. The biochemical and molecular properties of the traditional Chinese medicine monomers herein are summarized in Table 1.

### Combination with EGFR-TKIs to reverse resistance

**Figure 3:**
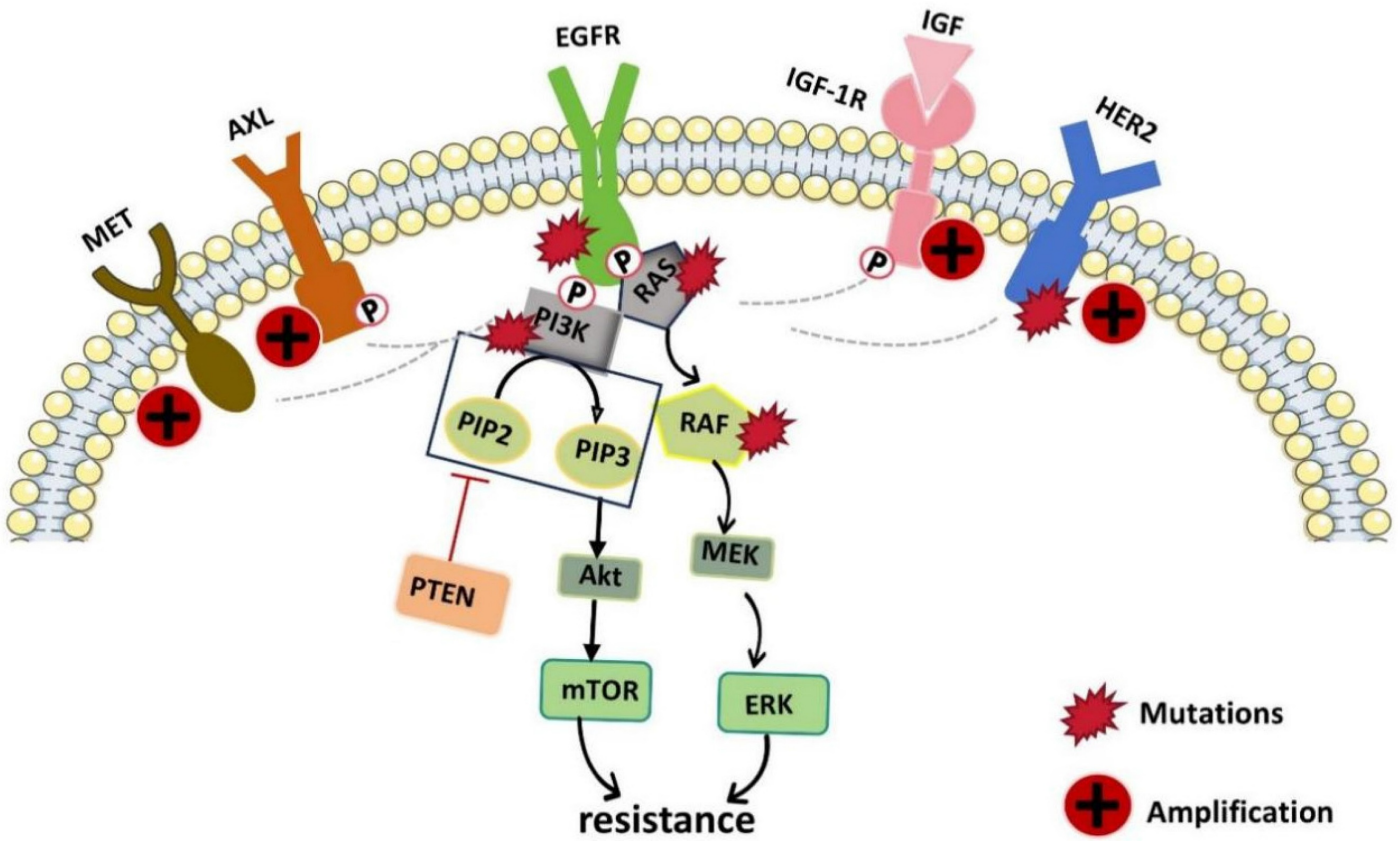
Schematic diagram of the molecular pathways related to EGFR-TKI resistance. EGFR kinase domain mutations and the upregulation or activation of other RTKs (such as AXL, MET, HER2 and IGF-1R which can cross the suppressed EGFR signaling pathway and activate downstream pathways) lead to resistance to EGFR-TKIs.

Although EGFR-TKIs have negligible side effects and satisfactory therapeutic efficacy, their treatment plans are hindered by almost inevitable drug resistance. When patients are administered EGFR-targeted therapy with significant early effects, drug resistance-related problems occur within only 9-12 months, which greatly hinders the subsequent treatment of tumors and the efficacy of drugs 76. Resistance to EGFR-TKIs is caused mainly by acquired EGFR mutations or EGFR-independent pathways, including gene mutations, bypass activation, histological transformation, overexpression of efflux pumps, increased metabolic inactivation, etc. (Fig. 3.) 77. Therefore, many researchers have investigated ways to reverse or delay this resistance. The use of traditional Chinese medicine monomers and traditional Chinese herbs as supplements is a promising strategy for reversing drug resistance. In this section, we described some of the common and more important TCMs that can reverse or prevent the development of tumor resistance to EGFRIs. The results are summarized in Table 2.

Osimertinib is the first approved third-generation EGFR-TKI that can effectively address the issue of acquired resistance associated with first-generation EGFR-TKIs, such as those that target acquired T790M mutations and other common EGFR activating mutations 78. Therefore, it is widely used in clinical practice. However, after about 10-19 months, drug resistance emerges which leads to uncontrolled cancer and further disease progression 79. Therefore, corresponding measures need to be developed to overcome acquired resistance to Osimertinib. Bufalin is a types of bufadienolides, and it is an effective ingredient in secretions of toads and in certain plants 80. Bufalin can induce apoptosis in various types of cancer cells 59. Prior research has demonstrated that bufalin can inhibit MCL-1 81. One important mechanism controlling the effectiveness of Osimertinib is U70-mediated overexpression of MCL-1 82. Cao et al., reported that bufalin and osimertinib together significantly increased the expression of cleaved-caspase-3 and PARP and induced apoptosis in osimertinib-resistant lung cancer cells compared to either medication alone. Therefore, bufalin can increase the sensitivity of Osimertinib-resistant lung cancer cells. The results of this study demonstrated a novel therapeutic combination that targets the Ku70/MCL-1 signaling axis to overcome osimertinib resistance 83. The main limitations include their low solubility, potential cardiotoxicity and rapid distribution and metabolism *in vivo*, which make their use in the clinic difficult.

Berberine is an isoquinoline alkaloid compound isolated from *Coptis chinensis* plants. Due to its antibacterial, anti-inflammatory, and metabolism-regulating qualities, berberine is used as a traditional Chinese medicine monomer. It has been studied as the main and complementary therapeutic technique for treating various diseases 61. It exhibits antitumor effects in many types of cancer, including breast cancer, lung cancer, and liver cancer. 84. Chen et al. reported that berberine, an inhibitor of MET, shows therapeutic potential when it is administered along with osimertinib or other third-generation EGFR-TKIs to overcome acquired resistance caused by MET amplification. *In vivo*, the growth of MET-amplified osimertinib-resistant tumors is more effectively inhibited when berberine and osimertinib are used together 85.

Luteolin, also known as 3',4',5,7-tetrahydroxylflavone, is a component of flavonoids. It is widely distributed in various fruits, vegetables, and herbs, such as honeysuckle and celery 62. It has anti-inflammatory, antitumor, and antioxidant effects on the intestinal flora and neuroprotective effects 62. Luteolin prevents the progression of various types of cancer 86. Owing to the synergistic effect of luteolin and PD-1 inhibitors, it can be used as an adjuvant therapy 87. Huang et al. reported that luteolin, in combination with osimertinib overcomes acquired resistance to osimertinib induced by MET amplification and overactivation by inhibiting the EGF-MET-Akt pathway 88. To prevent or reverse the resistance to EGFR-TKIs caused by MET amplification, whether it can be appropriately combined with berberine or luteolin should be considered. However, further validaion by many subsequent studies is necessary.

Polyphyllin I is isolated from the natural herb *Paris polyphylla*. PPI has anticancer potential 65. It can inhibit cisplatin-resistant NSCLC growth by regulating p53 and CIP2A/AKT/mTOR signal transduction 89. HIF-1 accelerates tumor growth and metastasis by regulating the metabolism and immune escape of tumor cells, and the level of this protein is elevated in gefitinib resistant lung cancer cells 90, 91. Zhang et al. reported that PPI can improve gefitinib resistance and inhibit the VEGF/VEGFR2/p38 pathway by targeting HIF-1α in lung adenocarcinoma 92. Lai et al. reported that PPI reversed resistance in osimertinib-resistant NSCLC cell lines and xenografts by regulating PI3K/AKT signaling and controlling the expression of apoptosis-related proteins. These findings suggested that PPI is a promising therapeutic agent for reversing osimertinib resistance 93. Thus, PPI can reverse gefitinib resistance and osimertinib resistance, and it might serve as a promising treatment for reversing resistance to EGFR-TKIs.

Rhein is an anthraquinone compound widely found in herbs such as rhubarb and is popular to for its anti-inflammatory and antitumor properties 70. Rhein is structurally similar to several STAT3 inhibitors 94. Many studies have reported that cancer drug resistance is closely related to the activation of STAT3 95, 96. Yang et al. conducted experiments and also tentatively confirmed our speculations. The results of *in vitro* experiments revealed that rhein and erlotinib, when used in combination, inhibited the phosphorylation of STAT3 and EGFR in pancreatic cancer cell lines, thereby inhibiting the proliferation of pancreatic cancer cells. Similarly, *in vivo* experiments confirmed this finding 97. Studies on colorectal cancer reached similar conclusions 98. The combination of rhein and EGFRIs may, therefore, be a novel antitumor strategy that can overcome resistance to EGFRIs and have a stronger antitumor effect.

TCM has played a significant role in reducing the toxicity of chemotherapy, enhancing immune function, and even directly treating and slowing the progression of cancer in recent years 99. A combination of TCM compounds can reverse resistance to EGFR-targeted drugs 99. Si Jun Zi Tang (SJZT) is a classic formula that has Qi tonifying properties and can manage the underlying causes of lung cancer, colon cancer, and stomach cancer 100, 101. SJZT combined with gefitinib has an antitumor effect on gefitinib-resistant (PC9-GR) cells. SJZT may affect glutamine metabolism by modulating key targets involved in glutamine metabolism (SLC1A5, GLS and GS) and regulating the levels of related metabolic markers, ultimately reversing gefitinib resistance 102. Therefore, SJZT may be used as an adjunct to gefitinib therapy to prevent or reverse resistance.

### Synergistic effects through other pathways

Owing to their biodiversity and structural complexity, TCMs can serve as drugs for multitarget therapy 103, 104. The efficacy of EGFR-targeted therapy, however, is often limited by the singularity of their targets. Therefore, multitargeted therapy for cancer to improve its efficacy has gained the attention of the medical community 105. The research, extraction, translation and clinical application of traditional Chinese medicine monomers or compound prescriptions for single-target treatment of cancer have not been solved. Therefore, its use as a complementary or adjuvant therapy in combination with EGFR-targeted treatment of cancer provides a new way to address these problems 106, 107. The following section describes several representative traditional Chinese medicine monomers or compound prescriptions that, when used along with EGFRIs for treating cancer, can have other synergistic effects, such as delaying or preventing metastasis and multitargeting effects to improve the efficacy and response to chemoresistant advanced tumors (Table 3).

Cancer treatment modalities have been updated and optimized, and the use of combinations of two or more compounds has gained greater attention. Epigallocatechin-3-gallate (EGCG) is a common active ingredient extracted from green tea and has anti-inflammatory, antibacterial and anticancer activities 71. EGCG has anticancer activities against various types of cancer, such as colorectal cancer, squamous cell carcinoma of the head and neck (SCCHN), and thyroid cancer 108. Abedul Haque et al. reported that the combination of EGCG and erlotinib induced the expression of the cell cycle regulatory proteins p21 and p27 and the apoptosis-regulating protein Bim and inhibited the expression of Bcl-2 109. Therapeutic techniques targeting EGFR remain the mainstay of treatment for advanced cancers, and this preclinical study may influence future treatment options for SCCHN.

Curcumin is a TCM monomers isolated from the rhizome of turmeric. It has anti-inflammatory, antioxidant, and anticancer effects 110. It is safe for pharmacological applications and has negligible toxicity with high efficacy, which can promote cancer cell apoptosis, antitumor proliferation, antitumor metastasis, etc. 111, 112. Curcumin can impede the EGFR signaling pathway in cancer cells by directly or indirectly inhibiting the enzymatic activity of the EGFR intracellular domains, inhibiting EGFR phosphorylation and inducing EGFR ubiquitination 113. It can also be used along with different chemotherapeutic drugs to cause apoptosis and block the proliferation of various tumor cells 114. Curcumin can also be used in combination with EGFRIs. Combination treatment increases the inhibitory effect of curcumin and cetuximab on survival and the induction of apoptosis in cisplatin-resistant oral cancer. Kuroda et al. found a close relationship between cisplatin resistance and the EGFR signaling pathway in lung cancer cells. These findings suggest that the use of EGFRIs may be a promising strategy for treating cisplatin resistance 115. Curcumin is, therefore, a possible adjuvant therapy, and the combination of curcumin and cetuximab may result in a novel treatment for oral cancer and even cisplatin resistance.

Shikonin is the main component of the Chinese herb *Lithospermum erythrorhizon* (zicao) 116. This pkm2 inhibitor has antitumor effects on different types of cancer 117. Tang et al. reported that gefitinib inhibits EGFR wild-type lung cancer cells more strongly when PKM2 is knocked down. Therefore, shikonin, a natural PKM2 inhibitor, significantly promoted apoptosis and inhibited the proliferation of EGFR wild-type lung cancer cells when combined with gefitinib. The results of *in vivo* and *in vitro* experiments confirmed this conclusion 118. EGFR TKIs were originally only targeted at a subset of the population, and such studies may help improve the efficacy of EGFR TKIs against EGFR wild-type tumors.

Platycodin D (PD) is the main triterpenoid saponin extracted from the roots of *Platycodon grandiflorum* 72. PD suppresses the lung metastasis of breast cancer by downregulating PD-L1 expression and enhances the anticancer effects of sorafenib in AKT-positive and PTEN-negative prostate cancer cells through the ubiquitination of p-Akt 119, 120. PD and cetuximab (CTX) can prevent colorectal cancer (CRC) cells from proliferating 121. When the two drugs are used in combination, the metastasis inhibition effect is the best, as both relatively unrelated pathways are inhibited 122. When individuals are clinically susceptible to the metastasis of CRC, we can pay attention to these TCM natural products, such as PD.

Modern Western medicine and TCMs have been combined in recent years to increase clinical efficacy and decrease painful responses. Additionally, EGFR-TKIs and the components of TCM can prolong patient survival. TCM drugs in conjunction with EGFR-TKIs can prolong patient survival and postpone drug resistance; they can be administered along with chemotherapy, targeted treatment, and prompt replacement of a new generation of TKI preparations 123.

Traditional Chinese medicine monomers and compounds can serve as potential drugs for multitarget therapy because of their biodiversity and structural complexity. The efficacy of EGFR-targeted therapeutic techniques, however, is often limited by the singularity of their targets. Therefore, multitargeted therapy for cancer to improve its efficacy has gained the attention of the medical community. The research, extraction, translation and clinical application of traditional Chinese medicine monomers and compounds for single-target treatment of cancer have not been solved; therefore, their use in complementary or adjuvant therapy in combination with EGFR-targeted treatment of cancer provides a new way to address these problems.

### Mitigation of related side effects

Although EGFRIs have relatively few side effects, many individuals experience symptoms, including diarrhea, alopecia, and skin toxicity 124. In addition to the application of antibiotics and topical ointments, Chinese herbal medicines can be used to alleviate severe skin diseases caused by EGFRI treatment 125. The following section describes several representative TCM monomers or compound prescriptions used clinically to alleviate the adverse effects induced by EGFR-targeted therapies (Table 4).

Honeysuckle is a traditional herb used to treat rashes and is safe for humans 126. A clinical study demonstrated that honeysuckle can effectively reduce the frequency and intensity of acne-like rash caused by EGFRIs, especially in preventive treatment. 127. An acne-like rash usually appears within the first 1-2 weeks after treatment with EGFRIs. The preventive treatment with honeysuckle showed promising efficacy in reducing the incidence and severity of EGFRI-induced acne-like rashes. The application of EGFRIs for treatment may improve preventive therapy 128.

Qi Yin San Liang San Decoction (QYSLS) (30 g of raw *Astragalus membranaceus*, 30 g of honeysuckle, 30 g of angelica, 10 g of raw liquor, and 1 g of centipede) has been used to treat immune-related disorders and adverse effects associated with tumor therapy 129. To construct a mouse model of adverse skin reactions, gefitinib was used to induce adverse skin responses in mice *in vivo*. Besides having a preventive effect, treatment with different doses of QYSLS can repair the immunological damage induced by gefitinib and reduce the incidence of cutaneous side effects. HaCaT cells were used as an *in vitro* adverse reaction model to validate the relevant active chemicals and targets. These findings showed that in HaCaT cells treated with gefitinib, the active components luteolin and quercetin, which were identified through network pharmacology and molecular docking techniques, increased the expression of PTGS2 and MMP9 and decreased the production of CCL2 130.

Pien Tze Huang is a TCM developed by ancient Chinese imperial physicians. Its main ingredients include musk, bezoar, snake gall, and Sanqi 131. Many studies have shown that this TCM can treat diseases such as tumors and inflammation 132, 133. Additionally, Pien Tze Huang can promote skin healing and alleviate many problems related to skin damage 134. An external ointment based on the traditional formula of Pien Tze Huang combined with oil-based ingredients has been developed. In clinical practice, PZH Unguentum Compositum was used to cure skin damage caused by erlotinib treatment. One patient developed a grade 2 rash after treatment with erlotinib and was clinically treated with PZH Unguentum Compositum. After two weeks, the symptoms were significantly relieved and had almost disappeared without any related side effects. 135. However, the relevant mechanism needs to be elucidated. In China, Pien Tze Huang, especially PZH Unguentum Compositum, is widely used and is anticipated to play a significant role in the treatment of EGFR-TKI-related rashes. However, further studies are needed on the therapeutic effects, related mechanisms, and clinical application plans.

Shenling Baizhu powder (SBP) is a classic Chinese medicinal compound. *In vivo* studies have shown that SBP can alleviate diarrhea caused by pyrrolitinib treatment 136. Although targeted therapy can achieve high efficacy, the dose needs to be reduced or treatment may even need to be stopped for some patients due to the occurrence of adverse reactions such as diarrhea. This has a strong negative effect on the therapeutic effect of cancer 137. On the other hand, SBP is safe and has strong therapeutic effects because of its long history. Therefore, some researchers speculate that SBP can alleviate diarrhea caused by targeted therapy 138. A prospective randomized controlled study using modified SBP confirmed this finding. The results suggested that modified SBP was better than imodium in improving the symptoms of diarrhea, inhibiting the recurrence of diarrhea, and improving the physical condition of patients.^139^. However, further clinical and basic studies are needed to elucidate the mechanism of action of modified SBP and to guide the clinical use of this drug.

To summarize, there is some evidence that TCMs, as a complementary or adjuvant therapy, can alleviate or even eliminate the adverse effects induced during EGFRI-based cancer treatment. However, only a few studies on this topic have been published, and the methodological rigor and precision of the studies are not adequate to draw definitive conclusions.

### Clinical evidence of combination therapy

Many researchers have stated the clinical importance of TCMs as stand-alone options for treating cancer. However, in most cases, TCMs are used as part of combination therapy owing to their bioavailability, metabolic instability, potential toxicity, and poor targeting ability. Many studies have emphasized its potential for use in combination with targeted therapy for treating cancer. Moreover, TCM is a patient-specific personalized treatment plan consisting of natural products with different pharmacological effects. Here, we summarized some findings of clinical trials demonstrating the clinical potential of combination therapy. Moreover, by analyzing the results of these clinical trials, we aimed to determine the opportunities provided by and challenges associated with natural products and personalized TCM treatments to further utilize their strengths and avoid their weaknesses for better treatment of patients with lung cancer.

The main active ingredient of ginseng known as Rg3 ginsenoside has antiangiogenic and antitumor activity 75. A clinical trial at Xinqiao Hospital of the Third Military Medical University investigated the clinical benefits of the combination of ginsenoside and an EGFR-TKI in the treatment of advanced non-small cell carcinoma in patients. The results revealed that the median PFS for patients treated with the combination therapy was 12.4 months (95% CI: 9.2-15.6), whereas it was 9.9 months (95% CI: 8.4-12.6) for patients treated with EGFR-TKIs alone. Their OS values were different but not statistically significant. 140. These results suggest that the addition of ginsenoside Rg3 to EGFR-TKI reduces drug resistance in the treatment of NSCLC patients with EGFR-active mutations, and that ginsenoside Rg3 could be an important agent for improving median PFS and delaying the acquisition of resistance to EGFR-TKI in patients.

In China, TCM is the most widely used complementary therapy. Fu Zheng Kang Ai Formula has been used as a complementary and alternative therapy for cancer treatment for several years 141. In a randomized controlled clinical trial, patients who met the inclusion criteria were randomly divided into the gefitinib + FZKA (GF) group and the gefitinib (G) group. The PFS of the GF group was 12.5 months (95% CI: 3.30-21.69), whereas that of the G group was 8.4 months (95% CI: 6.30-10.50). Additionally, the MST of the GF group was 21.5 months (95% CI: 17.28-25.73), whereas that of the G group was 18.3 months (95% CI: 17.97-18.63). These findings suggested that FZKA can prolong the survival of patients with advanced NSCLC treated with gefitinib 142. Some experimental results have shown that FZKA enhances the apoptosis of lung cancer cells induced by gefitinib through the mitochondrial pathway. These findings suggested a new molecular mechanism by which FZKA delays drug resistance in the treatment of patients with lung cancer 143.

A cohort study found that mPFS was 12.3 months for patients treated with TCM and EGFR-TKI, compared to just 8.9 months for patients treated with EGFR-TKI monotherapy. The results also showed that patients in the combination therapy group had an mOS of 28.2 months compared to 24.2 months in the monotherapy group. The TCM treatments administered are tailored to each patient's specific symptoms, ensuring a personalized approach to care 144. EGFR-TKIs combined with TCM as first-line therapy resulted in longer mPFS than EGFR-TKIs alone and significantly prolonged patient survival.

The three studies examined the efficacy of combining EGFR-TKI with TCM in patients with cancer, with PFS as the primary endpoint. Due to the small sample size of the experiments, it was not sufficient for OS analysis. And the existing results showed that there was no statistically significant difference in OS. All three studies demonstrated a significant improvement in PFS with EGFR-TKI + TCM compared to EGFR-TKI alone. Despite clinical heterogeneity (TCM composition, study design), all studies consistently showed PFS benefit. The HRs < 1 and P-values < 0.05 indicate a statistically significant reduction in progression/death risk with combination therapy.

### Safety Considerations and Adverse Effects

While the combination of TCM monomers and compound prescriptions with EGFRIs offers promising therapeutic benefits, the adverse effects of these combined regimens need to be considered. Many natural products and TCM compounds can interact with EGFRIs, altering their pharmacokinetics or pharmacodynamics. For example, curcumin, an anti-inflammatory and anticancer agent, inhibits cytochrome P450 enzymes, which may increase the plasma concentrations of EGFR-TKIs and exacerbate their side effects 145. Similarly, berberine, which can effectively overcome MET-induced resistance, may cause gastrointestinal discomfort, potentially worsening the diarrhea commonly associated with EGFRIs 146.

Some TCM monomers, such as bufalin and PPI, have intrinsic toxicity that may cause problems when combined with EGFR-TKIs. Bufalin, derived from toad venom, has a narrow therapeutic window and may cause cardiotoxicity at high doses 147. Rhein, while effective in reversing resistance, may also cause hepatotoxicity or nephrotoxicity, especially when combined with EGFR-TKIs that already have hepatotoxic effects 70.

Certain TCM formulae, such as the Fu Zheng Kang Ai Formula, are designed to enhance immune function, which may increase the risk of immune-related adverse events when combined with EGFRIs. Additionally, while TCM formulae such as modified SBP can alleviate EGFRI-induced diarrhea, their long-term safety and potential for additive gastrointestinal toxicity require further investigation.

In conclusion, while the combination of TCM monomers and compound prescriptions with EGFRIs holds great promise, the potential adverse effects need to be considered. Future studies should focus on dose optimization, patient-specific factors, and rigorous safety evaluations to ensure the safe and effective use of these combined regimens in clinical practice.

### Cost-effectiveness

Evaluating the economic viability of combining TCM monomers and compound prescriptions with EGFRIs will be critical for widespread adoption, especially in resource-limited settings.

One of the primary advantages of combining TCM monomers and compound prescriptions with EGFRIs is the ability to reduce drug resistance and mitigate side effects, both of which contribute to significant cost savings. Natural products such as bufalin and berberine can reverse resistance to EGFR-TKIs, such as osimertinib, thus extending the duration of effective treatment 83, 85. This reduces the need for costly second-line therapy or experimental drugs, which are often associated with higher prices and limited efficacy. Moreover, by reducing the severity of these side effects, these therapeutic methods can decrease the need for additional medications, hospital visits, and supportive care, reducing overall treatment costs.

TCM monomers or compound prescriptions often exhibit multitarget effects, which can increase the efficacy of EGFRIs while reducing the need for additional targeted therapy. This synergistic effect may reduce the need for combination therapy with other expensive targeted agents, thereby making the treatment more cost-effective.

Although TCM monomers and compound prescriptions are generally perceived as inexpensive because of their relatively low production costs, several factors must be considered. The production of high-quality, standardized TCM natural products or compound prescriptions requires rigorous quality control measures, which can increase costs. Most natural products have poor oral bioavailability, poor intestinal permeability, and high toxicity, necessitating the use of higher doses or advanced delivery systems to achieve therapeutic effects. These modifications can increase production costs but may still be more economical than targeted therapy.

The combination of TCMs with EGFRIs is a promising strategy to increase therapeutic efficacy, reduce drug resistance, and mitigate side effects. While these treatment techniques can decrease costs through reduced treatment durations, fewer side effects, and improved patient outcomes, their economic viability depends on factors such as production costs, bioavailability, and long-term clinical benefits.

## Discussion

This review summarizes some TCM monomers and compound prescriptions that can be used in combination with EGFR inhibitors. It is found that the enhancement of the therapeutic effect on cancer mainly occurs through three aspects: reversing drug resistance, generating synergistic effects, and alleviating adverse reactions. Moreover, some clinical trials have verified that the combination has produced good effects on patients. At the same time, we have also summarized the biochemical and molecular characteristics of these TCM monomers and found that they all have poor bioavailability and certain toxic effects and other features. Therefore, several challenges must be addressed to translate these preclinical findings into clinical practice. How to improve bioavailability and the cost brought by the process of improving its utilization are also issues that need to be considered and solved urgently. At the same time, we have to consider whether more serious toxic and side effects will occur when combined, which brings great difficulties to clinical application.

### Mechanistic Insights and Therapeutic Potential

TCM monomers and compound prescriptions exhibit diverse mechanisms of action, including inhibition of EGFR-related signaling pathways, reversal of drug resistance, and mitigation of side effects. For instance, bufalin and berberine have shown efficacy in overcoming osimertinib resistance by targeting the Ku70/MCL-1 axis and MET amplification, respectively. Similarly, luteolin and polyphyllin I synergize with EGFR-TKIs to inhibit cancer cell proliferation and metastasis. These findings underscore the multitargeted nature of TCMs, which could complement the singular focus of EGFRIs and improve therapeutic outcomes.

### Clinical Evidence and Practical Applications

Clinical studies support the adjunctive use of TCM monomers or compound prescriptions with EGFRIs. For example, ginsenoside Rg3 and Fu Zheng Kang Ai Formula have demonstrated the ability to prolong progression-free survival (PFS) and overall survival (OS) in patients with non-small cell lung cancer (NSCLC). Additionally, TCM formulations like Qi Yin San Liang San Decoction and Pien Tze Huang have been effective in alleviating EGFRI-induced skin toxicity and diarrhea. These results highlight the potential of TCMs to enhance patient quality of life and treatment adherence.

### Challenges and Limitations

#### Bioavailability and Toxicity

The poor bioavailability of many TCM monomers represents a major translational barrier. Most bioactive compounds exhibit limited aqueous solubility, rapid metabolic clearance, and inefficient systemic absorption 148. For example, curcumin's extensive first-pass metabolism and rapid conjugation in the liver and intestinal wall result in negligible systemic availability 149. Similarly, berberine's high affinity for P-glycoprotein efflux pumps and extensive phase II metabolism severely limit its oral bioavailability 150. These pharmacokinetic shortcomings often necessitate the administration of supraphysiological doses to achieve therapeutic effects, which in turn increases the risk of adverse reactions.

Addressing these challenges requires innovative formulation strategies and careful clinical monitoring. Advanced drug delivery systems, including nanoparticle encapsulation and lipid-based formulations, have shown promise in improving the bioavailability of problematic compounds while potentially mitigating their toxicity 151. Furthermore, the development of structurally modified analogs with optimized pharmacokinetic profiles may offer a path forward. However, these approaches must be balanced against the potential loss of therapeutic efficacy that sometimes accompanies structural modification of TCM monomer scaffolds.

#### Standardization and Quality Control

The clinical integration of Traditional Chinese Medicine (TCM) and natural product formulations faces significant challenges due to their inherent complexity and variability. Unlike single-compound pharmaceuticals, TCM preparations typically contain hundreds of bioactive constituents that interact in complex, often poorly understood ways.

Modern standardization efforts must therefore develop innovative strategies that respect the integrity of traditional formulations while meeting contemporary pharmaceutical standards. This requires moving beyond simple chemical fingerprinting to develop more sophisticated systems of characterization that can capture the essential therapeutic qualities of these complex mixtures. The challenge lies in identifying which aspects of this complexity are therapeutically essential and which can be standardized without compromising efficacy.

#### Drug Interactions

While the combination of TCM monomers and compound prescriptions with EGFRIs holds great promise, the potential adverse effects need to be considered. Many natural products and TCM compounds can interact with EGFRIs, altering their pharmacokinetics or pharmacodynamics. Future studies should focus on dose optimization, patient-specific factors, and rigorous safety evaluations to ensure the safe and effective use of these combined regimens in clinical practice.

## Future Directions

### Optimizing Delivery Systems

The development of advanced delivery systems represents a critical pathway for overcoming the pharmacological limitations of TCM monomers in cancer therapy 152. Current research focuses on creating innovative formulations that can simultaneously address poor bioavailability, enhance tumor targeting, and minimize systemic toxicity. Nanotechnology-based approaches have emerged as particularly promising, with various nanoparticle platforms being explored to protect bioactive compounds from premature degradation and improve their pharmacokinetic profiles. Surface modification strategies, such as PEGylation or the conjugation of targeting ligands like EGFR antibodies, offer the potential for tumor-specific accumulation while reducing off-target effects. The integration of these delivery technologies must be carefully balanced with preserving the biological activity of complex natural product mixtures, particularly for TCM formulations where multiple active components may require co-delivery.

### Personalized Medicine

Tailoring TCM formulations to individual patient profiles could maximize therapeutic benefits while minimizing adverse effects. Ongoing research focuses on identifying verifiable biomarkers that bridge TCM diagnostic patterns with modern molecular pathology, enabling truly evidence-based personalization. When fully realized, this approach promises to enhance EGFR-TKI efficacy while maintaining the holistic benefits of TCM and minimizing herb-drug interaction risks.

### Efficacy and Cost Benefits

The combination of TCMs with EGFRIs is a promising strategy to increase therapeutic efficacy, reduce drug resistance, and mitigate side effects. While these treatment techniques can decrease costs through reduced treatment durations, fewer side effects, and improved patient outcomes, their economic viability depends on factors such as production costs, bioavailability, and long-term clinical benefits.

## Conclusion

EGFR is one of the most promising and appealing clinical and scientific targets in cancer therapy, and EGFR-targeted therapy is considered to be a potential anticancer treatment strategy. The groundbreaking advancements in cancer treatment have been greatly aided by EGFR TKIs and mAbs. For example, some options limit the scope of use to cancer types with the overexpression of EGFR, and most patients become resistant to therapy within a year of starting treatment, leading to severe side effects, including rashes. These changes significantly decrease the effectiveness of medications. To increase antitumor effectiveness, more potent, safe, and inexpensive supplements, synergists, or additional anti-EGFR medications should be developed.

TCMs are a potential therapeutic approach to address these issues because of their diverse functions, including but not limited to blocking EGFR-related signaling pathways, sensitizing EGFR-targeted drugs, reversing resistance to EGFR-targeted drugs, and alleviating the side effects of targeted therapy. Owing to the unique pharmacological and biological activities of traditional Chinese medicine monomers, drug development based on the structure and properties of their components occupies an important position in the field of contemporary new drug development. Numerous traditional Chinese medicine monomers have been used in recent years for targeted EGFR therapy. Most of these drugs are only used as a pretreatment or supplement to EGFR-TKIs and mAbs, and studies on this topic are in their early stages. Several issues still need to be resolved. There are major obstacles to its widespread use in clinical practice due to its limited oral bioavailability, poor biological activity, severe toxic side effects, and limited supplies. Owing to the complexity of the components of natural compounds, in-depth studies on their pharmacological targets and mechanisms of action are challenging, which makes their optimal application in cancer therapy difficult to implement. Owing to the complexity of their pharmacology and individual differences, it is challenging to standardize the study of Chinese herbal remedies. Many traditional Chinese medicine monomers, such as curcumin and berberine, which have been extensively studied for their anticancer properties, can be used for EGFR-targeted therapy. Curcumin is very safe in humans, even at high doses, but its low bioavailability greatly limits its clinical use 69. Berberine is also a promising traditional Chinese medicine monomer, but its anticancer efficacy is greatly hindered by its low bioavailability and water solubility, as well as its tendency to induce allergic reactions when it is injected intramuscularly and intravenously 61. Therefore, better utilization of these TCMs is needed to treat cancer.

Natural products, especially many TCM products or their compound formulae that have a unique clinical application history and foundation in China should be continuously innovated using science and technology. These products should be combined with modern medicine to realize the innovation of Chinese medicines, the advancement of the pharmaceutical industry, and the health and well-being of people.

With continuous in-depth research on EGFR, TCMs that target EGFR will have greater potential for utilization in cancer treatment.

## Figures and Tables

**Table 1:** Biochemical and molecular characterization of traditional Chinese medicine monomers.

Name	Chemical class	PKDM	solubility	Toxicity	Mechanism of Action	Bioactivity	Ref
Bufalin	steroid	Low bioavailability; Excreted mainly through both hepatic pathway and renal pathway.	Insolubility in water; Good solubility in organic solvent	Endogenously expressed in humans and should not cause toxicity if used at physiologic doses	Acting on Na/K-ATPase pump; Promotion of cell cycle arrest and apoptosis.	harbouring cardiotonic, anti-inflammatory, and diuretic properties, anti-cancer.	58, 59
Berberine	alkaloid	Low bioavailability (0.37 ± 0.11%); Excreted mainly via fecal and renal excretion route.	Low solubility and poor permeability. Berberine sulfate is usually more water-soluble and stable	High doses or prolonged use may cause toxic reactions.	Inhibition of DNA topoisomerase, Induction of reactive oxygen species production	antioxidant, anti-microbial, antiinflammatory, anti-cancer, anti-diabetes, anti-dyslipidemia, and anti-obesity	60, 61
Luteolin	flavonoid	Low bioavailability; The majority of luteolin possibly be metabolized to other compounds, and these metabolites could then absorb into the systemic circulation or return to the small intestine through the enterohepatic circulation.	Low solubility in water	Nontoxic side effects as a dietary supplement.	Inhibition of ROS-induced DNA, lipid, and protein damage, induction of apoptosis and cell cycle arrest, inhibition of metastasis and angiogenesis.	Anti-microbial, anti-inflammatory, anti-allergy, anti-oxidant, and anti-cancer.	62, 63
Polyphyllin I	Saponins	Low oral bioavailability (about 0.62%);	Low solubility in water		Activation of the JNK signaling pathway, inhibition of PDK1/Akt/mTOR signaling, induction of autophagy, cell cycle arrest and apoptosis	Anti-fungal, anti-cancer, anti-inflammatory, anti-oxidant	64, 65
Shikonin	naphthoquinone	Low bioavailability; metabolized primarily in the liver, involving the CYP450 and UGT enzyme systems, and excreted in urine and bile.	Insolubility in water; Good solubility in alkaline substances and organic solvent	May cause toxicity, especially in drug interactions based on effective inhibition of CYP enzymes.	Inhibition of PKM2 TNF-α and NF-κB pathway	Anti-inflammatory, anti-cancer, cardiovascular protective, antimicrobial,	66, 67
Curcumin	phenolic	Low bioavailability; Absorption in the small intestine is low and rapidly eliminated in the liver	Poor solubility in water at physiological pH	prescribed doses of curcumin have no side effects in the body	Inhibition of NF-κB, p300/CREB-binding protein-specific	Antioxidant, anti-inflammatory, anti-infective, antiangiogenic and anti-cancer	68, 69
Rhein	lipophilic anthraquinone	Low bioavailability The Rhine absorbed into the body is first metabolized into forms of glucuronide and sulfate, and then excreted into the hepatoenteric circulation through bile.	Insolubility in water; Good solubility in alkaline substances	Mainly related to the concentration and duration of administration. Low-dose short-term treatment has a protective effect on the liver and kidneys	Regulation of PI3K/AKT/ERK pathways, interference with NF-κB signaling pathway.	Hypoglycemic, lipid-lowering, anti-cancer, anti-inflammatory, anti-fibrotic, craniocerebral protection, antibacterial, and antiviral	70
epigallocatechin-3-gallate (EGCG)	polyphenol	Low bioavailability Poor intestinal stability and Low intestinal absorption. Excreted mainly via fecal, renal and air excretion route	Medium solubility in water, high solubility in acidic conditions	High doses or prolonged use may pose risks of liver toxicity, gastrointestinal discomfort, and drug interactions	Inhibition of cell proliferation and glutamate dehydrogenase 1/2 (GDH1/2, GLUD1/2) activity, induction of apoptosis,	Antioxidative, anti-inflammatory, anti-viral, anticancer, anti-microbial, anti-obesity and neuroprotective.	71
Platycodin D	Triterpenoid saponin	Low bioavailability (1.89%); Following intravenous and oral administration, small amounts of unchanged PD are excreted via the urinary and biliary (or fecal) routes	Good solubility in water.	Intravenous saponins may cause hemolysis.	Induction of apoptosis and autophagy in tumor cells, activation of AMPKα, inhibition of NF-κB pathway	Anti-viral, anti-oxidation, anti-tumor, anticoagulant, spermicidal, anti-inflammatory.	72, 73
ginsenoside Rg3	saponins	Low bioavailability easily transformed to active ingredients by gut microbiota	Poor solubility in water	Lower toxicity and higher safety	Targeting hypoxia-induced multiple signaling pathways, inhibition of NF-κB and MAPK pathway	Antioxidant, anti-inflammatory, antiangiogenic and anti-cancer	74, 75

**Table 2:** Traditional Chinese medicine monomers or compound prescriptions used in combination with anti-EGFR agents to reverse resistance.

Name	Origins	Structure	EGFRIs	Research Object		Effects (Target)	Ref
				*In vitro*	*In vivo*		
Bufalin	Chan Su	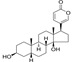	Osimertinib	NSCLC cells (PC-9/OR and HCC827/OR)	PC-9/OR xenograft mouse	Targeting the Ku70/MCL-1 signaling axis	83
Berberine	Coptis chinensis plant	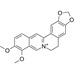	Osimertinib	EGFRm NSCLC cells (HCC827/AR)	HCC827/AR xenograft mouse	MET	85
Luteolin	Honeysuckl celery	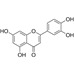	Osimertinib	NSCLC cells (H1975/OR)		MET	88
Polyphyllin I	Paris polyphylla	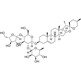	Osimertinib	NSCLC cells (PC-9/OR and H1975/OR)	H1975/OR xenograft mouse	PI3K/Akt	93
Polyphyllin I	Paris polyphylla	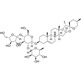	Gefitinib	NSCLC cells (PC9 and PC9/GR)	PC9/GR xenograft mouse	HIF-1a	92
Rhein	herbs such as rhubarb	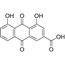	erlotinib	Human pancreatic cancer cells (BxPC-3, PANC-1, Patu8988T and AsPC-1)	BxPC-3 and PANC-1 xenograft mouse	STAT3	97
Sijunzi Tang (SJZ)			Gefitinib	NSCLC cells (PC-9/GR)	PC-9/GR xenograft mouse	SLC1A5, GLS and GS	102

**Table 3:** TCM monomers or compound prescriptions used in combination with anti-EGFR agents to produce synergistic effects through other pathways.

Name	Origins	Structure	EGFRIs	Research Object		Effects (Target)	Ref.
				*In vitro*	*In vivo*		
epigallocatechin-3-gallate (EGCG)	green tea	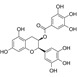	erlotinib	SCCHN cells (Tu686, Tu212)		Bim, Bcl-2	109
Curcumin	Curcuma longa	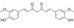	cetuximab	oral cancer cells (CAR)		EGFR	115
Shikonin	Lithospermum erythrorhizon (zicao)	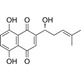	gefitinib	NSCLC cells (A549, H1299, H1975, HCC827)	A549 xenograft mouse	PKM2	118
Platycodin D	platycodon grandiflorum	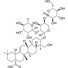	Cetuximab	CRC cells (HT29 and CaCo2)	HT29 and CaCo2 xenograft mouse	β-catenin	122

**Table 4:** TCM monomers or compound prescriptions used in combination with anti-EGFR agents to alleviate related side effects.

Name	structure	EGFRIs	Research Object		Side effects	Effects (Target)	Ref.
			*In vitro*	*In vivo*			
Honeysuckle		Gefitinib		Clinical research	acneiform rash		128
Qi Yin San Liang San Decoction	30 g of raw *Astragalus membranaceu*s, 30 g of honeysuckle, 30 g of angelica, 10 g of raw liquor, and 1 g of centipede	Gefitinib	HaCaT cell	a mouse model of adverse skin reactions	Skin adverse reactions and immune damage	PTGS2, MMP9 and CCL2	130
Pien Tze Huang	musk, bezoar, snake gall, and Sanqi	erlotinib		a case report	skin damage caused by erlotinib		135
Modified Shenling Baizhu powder (SBP)	Baibiandou, Lianzi, Dangshen, Fuling, Yiyiren, Chenpi, Baizhu, Jiegeng, Zhigancao, Sharen and Shanyao	EGFR-TKIs		Clinical research	diarrhea		139
